# Multilineage-differentiating stress-enduring cells alleviate atopic dermatitis-associated behaviors in mice

**DOI:** 10.1186/s13287-021-02671-5

**Published:** 2021-12-20

**Authors:** WenDi Fei, JunLin Wu, MengDie Gao, Qian Wang, Ya Yu Zhao, ChunLi Shan, Yu Shen, Gang Chen

**Affiliations:** 1 Key Laboratory of Neuroregeneration of Jiangsu and the Ministry of Education, Co-Innovation Center of Neuroregeneration, Nantong University, Nantong, 226001 Jiangsu Province China; 2 Center for Basic Medical Research, Medical School of Nantong University, Nantong, 226001 Jiangsu Province China; 3 Department of Anesthesiology, Affiliated Hospital of Nantong University, Nantong, 226001 Jiangsu Province China; 4 Department of Dermatology, Affiliated Nantong Hospital 3 of Nantong University, Nantong, 226001 Jiangsu Province China; 5 Medical School of Nantong University, Co-Innovation Center of Neuroregeneration, Nantong University, Nantong, 226001 China

**Keywords:** Muse cells, Pruritus, DNFB, Spinal cord, Atopic dermatitis, Glial cell

## Abstract

**Background:**

Pruritus is a recurring, long-lasting skin disease with few effective treatments. Many patients have unsatisfactory responses to currently available antipruritic treatments, and effective therapeutics are urgently needed to relieve symptoms. A previous study reported that mesenchymal stem cell (MSC)-mediated immune regulation could be used to treat skin inflammatory diseases. Multilineage-differentiating stress-enduring (Muse) cells are a new type of pluripotent stem cell that may also have the potential to treat inflammatory skin diseases.

**Methods:**

Muse cells were isolated from human bone marrow-derived MSCs (BMSCs) via the 8-h longterm trypsin incubation (LTT) method. Repeated use of 2,4-dinitrofluorobenzene (DNFB) induced atopic dermatitis (AD) in a mouse model. Immunofluorescence, behavior recording, and image analysis were used to evaluate the therapeutic effect of subcutaneous Muse cell injection. Real-time quantitative polymerase chain reaction (qPCR) was used to measure the expression of inflammatory factors. In vitro, wound healing and cell proliferation experiments were used to examine the effect of Muse cell supernatant on keratinocytes.

**Results:**

Our results showed that subcutaneous injection of Muse cells after AD model induction significantly alleviated scratching behavior in mice. The evaluation of dermatitis and photos of damaged skin on the back of the neck revealed that Muse cells reduced dermatitis, playing an active role in healing the damaged skin. The activation of spinal glial cells and scratching behavior were also reduced by Muse cell injection. In addition, we also showed that the expression levels of the inflammatory factors interleukin (IL)-6, IL-17α, and IL-33 in both the spinal cord and skin were suppressed by Muse cells. Furthermore, Muse cells not only exerted anti-inflammatory effects on lipopolysaccharide (LPS)-induced human HaCat cells but also promoted wound healing and keratinocyte proliferation.

**Conclusions:**

In vivo, Muse cells could alleviate scratching symptoms, reduce epidermal inflammation, and promote wound healing. In vitro, Muse cells could also promote the migration and proliferation of keratinocytes. In summary, Muse cells may become a new therapeutic agent for the treatment of AD.

## Background

Pruritus is a frequent symptom of many systemic and skin diseases. The International Forum for Itching Research (IFSI) defines itching that lasts 6 weeks or longer as chronic pruritus (CP). Due to the severity of CP and the fact that it is often difficult to treat, CP causes a heavy burden on individuals and impairs quality of life. In the patient population, the incidence of CP depends on the underlying cause of the disease. The overall incidence ranges from 25% in hemodialysis patients to 100% in patients suffering from skin diseases such as atopic dermatitis (AD) and urticaria [1]. AD is a complex inflammatory cutaneous disorder characterized by dysfunction of the epidermal barrier and immune dysregulation. Due to skin lesions, the activity of type 2 immunity in the skin causes the infiltration of cells that are mainly characterized by CD4 expression, and these cells interleukin (IL)-4 and IL-13, causing a cascade inflammatory reaction. The pathogenesis of AD is complex and seems to arise from a combination of environmental and genetic factors [2, 3]. There are several treatment approaches for AD, such as topical anti-inflammatory therapies, barrier repair, maintenance therapy, and oral Janus kinase (JAK) inhibitors. These therapies can only temporarily relieve symptoms, and they also cause side effects during longterm treatment [4]. Therefore, there is an urgent need to develop safe and effective treatments for AD.

Mesenchymal stem cells (MSCs) are nonhematopoietic progenitor cells derived from the stroma. MSCs are commonly present in various tissues in adults and newborns and can be expanded from the following tissues: bone marrow, umbilical cord blood, adipose tissue, dental pulp and skin [5]. In recent years, the immunomodulatory effect of MSC therapy has been confirmed in animal models, and this treatment can improve clinical symptoms by inhibiting the activation of T cells and B cells. MSC-mediated immune regulation can be used to treat skin inflammatory diseases. Current articles report that bone marrow-derived MSCs (BMSCs) have significant protective and therapeutic effects on ovalbumin-induced AD in mice by inhibiting IgE and IL-4 [6]. In stem cell-based therapy, there are some important issues that still need to be considered. MSCs are a mixed group of cells containing multiple subtypes. Researchers are still unclear about which type of cells have therapeutic effects. Therefore, it is difficult to establish MSC therapy guidelines that meet clinical treatment standards. During the process of repeated adherent culture, MSCs spontaneously differentiate, and after multiple passages, MSCs change their chemokines, cytokines, and receptors. In addition, the number of transplanted stem cells, pretreatment of the cell preparation, effective treatment approaches, and the frequency management also need to be considered [5]. Because of these limitations, MSCs are not suitable as a longterm treatment. Therefore, obtaining safe and stable cells is the key to promoting the clinical translation of research on stem cell treatment for AD.

Multilineage-differentiating stress-enduring (Muse) cells are a new type of pluripotent stem cell found in BMSCs and represent approximately 1%-2% of BMSCs. Muse cells have stable characteristics, form single cell-derived clusters in suspension, and maintain a single morphology [7]. Muse cells are endogenous pluripotent-like stem cells with stress-resistant and nontumorigenic characteristics that can spontaneously differentiate into multiple cell types to replace damaged cells and mediate tissue repair [7, 8]. In addition, Muse cells also have another important and unique feature: allogeneic and xenogeneic Muse cells escape host immune rejection after administration without the need for immunosuppressive therapy [9]. Based on these unique characteristics, intravenously administered allogenic Muse cells are already widely used in clinical trials [10]. At present, studies on Muse cells have focused on their differentiation ability, which is regarded as a new development direction for stem cell therapy [11–15]. However, the therapeutic efficacy of Muse cells in AD has not been reported. Therefore, we examined the antipruritic ability of Muse cells to provide a new direction for the clinical treatment of chronic itch.

## Methods

### Animals

Adult ICR mice (male, 25 g) were purchased from the Experimental Animal Center of Nantong University. Experiments are carried out according to the care and use guidelines on laboratory animals. The animals are kept in a dark-dark cycle of 12:12 h room, with free access to water and food.

### Drugs and administration

**Fig. 1:**
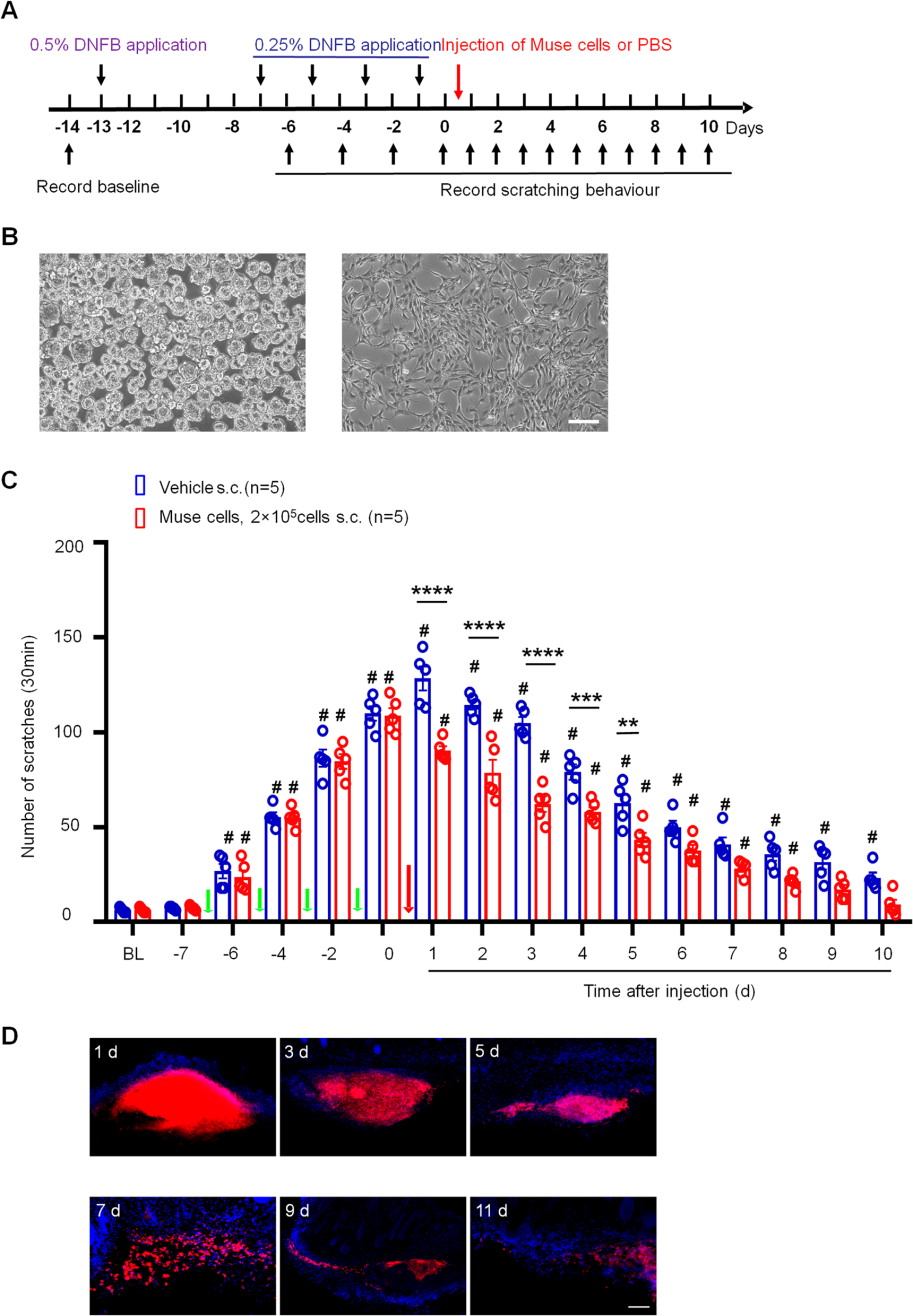
Pruritus in the back skin caused by 2,4-dinitrofluorobenzene (DNFB) was attenuated by Muse cells. **A** DNFB model time course. Mice were recorded to establish a baseline the day before 50 μl of 0.5% DNFB was applied to the abdominal skin. Five days later, 30 μl of 0.25% DNFB was administered to the skin of the nape of the neck and was further applied on the 3rd, 5th, and 7th days. The day after the last administration, Muse cells or PBS was injected into the back skin of the mice. The black arrows in this diagram indicate the time points at which scratching behavior were recorded. **B** Muse cell suspension culture (left) and adherence culture (right). Scale bar = 100 μm. **C** The bar graph shows the scratching behavior of mice administered DNFB with and without subcutaneous injection of Muse cells. On day 1–5, the number of scratches in the Muse cell-treated group was significantly reduced compared with that in the PBS group. Muse cell treatment quickly reversed the itching and scratching times. One-way or Two-way ANOVA followed by the Bonferroni test; #*p* < 0.05 versus BL; ***p < 0.001, **p < 0.01, *p < 0.05 versus vehicle group; *n* = 5 mice per group. The data are presented as the mean ± s.e.m. The green arrows indicate the time of DNFB painting and the red arrow indicates the time point of Muse cells or PBS injection. **D** The cultured suspension of dyed Muse cells was injected subcutaneously into the damaged skin. Day 1, 3, 5, 7, 9 and 11 post injection, the mice were sacrificed and skin tissues were collected. The number of Dil-labeled Muse cells (red) gradually declined, and only a few cells remained on day 11 after injection. Scale bar = 100 μm

To establish a model of atopic dermatitis (AD), we used 2,4-dinitrofluorobenzene (DNFB, Sigma-Aldrich) on the back skin of mice. Two days before sensitization, the hair on the abdominal surface and the neck was shaved. Dissolve the DNFB into an acetone and olive oil mixture (4:1). Using 50 μl of 0.5% DNFB to sensitize the shaved abdominal skin of mice. Five days later, 30 μl of 0.25% DNFB was challenged to the skin of the nape of mice, and then on the 3rd, 5th, and 7th day, the schematic diagram of the animal model is shown in Fig. 1A [16]. On the 8th day, and the next 7 days, the scratching behavior was recorded by video for 30 min. Then blindly count the number of scratches.

### Skin wound model

Under isoflurane anesthesia, the hair on the back of the neck of the mouse was shaved and a 5-mm long, full-thickness incision without injuring the underlying muscles was made by a blade and left unsutured. 2 × 10^5^ Muse cells were suspended in 20 μl PBS were injected into the subcutaneous tissue near the right side of the incision 1 day after surgery. The vehicle group received 20 μl PBS as control. Images were taken with a camera, and the area of the wound was analyzed with Image J.

### Neck injection of Muse cells

To study the effect of Muse cells on the itching caused by DNFB, 2 × 10^5^ Muse cells were dissolved in phosphate buffer saline (20 μl) and administered by subcutaneous injection after the last day painting of DNFB, the mice were sacrificed on day 1, 3, 5, 7, 9, and 11 after injection. The vehicle group received excipients (only phosphate buffer injection) on the same schedule.

### Behavioral analysis

All behavior records use double-blind testing. Before videoing, mice were acclimated to the testing environment for at least 2 days. On the day of the behavioral test, place the mice in a small plastic room on the elevated metal mesh floor and allow them to acclimate for at least 30 min. Then the mice's behavior was recorded for 30 min. Play the video offline when counting. The mouse raised its hind paw to scratch the back skin and put the paw back on the floor or mouth, it was regarded as a scratching behavior [17].

### Evaluation of dermatitis

Severity of dermatitis of the face, ears, and rostral part of the body was assessed by the following table. The total score (minimum 0, maximum 12) was expressed as the sum of each score of the four symptoms [18].

**Table:** 

Observation area	Score			
Face, ears, and the skin of neck	No symptoms (score 0)	Mild (score 1)	Moderate (score 2)	Severe (score 3)
Erythema/hemorrhage of the back skin	No erythema hemorrhage	Local erythema no hemorrhage on back skin	Disseminated erythema, no hemorrhage	Erythema on the entire back skin or Hemorrhage caused by repeated scratching
Edema in the ear pinna	No increase in ear thickness	Slight increase in thickness in either the left or right ear pinna	Marked increase in thickness of both sides of ear pinna	Marked increase in thickness and stiffness of both sides of ear pinna
Excoriation/erosion in the ear pinna	No excoriation and tissue deficit	Local (not continuous) excoriation, no tissue deficit	Small scale continuous excoriation, no tissue deficit	Continuous excoriation and tissue deficit
Scaling/dryness of the rostral back skin	No scaling or dryness	Local scaling and slight exfoliation of skin	Disseminated scaling/marked exfoliation of skin	Scaling of the entire area and marked exfoliation of skin

### Real-Time quantitative PCR (qPCR)

Cervical spinal cord, the skin of neck and HaCat cells were collected in RNase-free condition sand isolated total RNAs using TRIzol reagent (Sigma-Aldrich). RNA (1 mg) was reverse transcribed for each sample using SuperScript III RT (Vazyme). The sequences of the forward and reverse primers for:

- IL-6: Forward: TCCATCCAGTTGCCTTCTTGG; Reverse: CCACGATTTCCCAGAGAACATG;
- IL-17α: Forward: AGTGTTTCCTCTACCCAGCAC; Reverse: GCCACTGCCTCGTATTGAGT;
- IL-33: Forward: TCCTTGCTTGGCAGTATCCA; Reverse: TGCTCAATGTGTCAACAGACG;
- IL-1β: Forward: TGTCTTGGCCGAGGACTAAG; Reverse: TGGGCTGGACTGTTTCTAATG;
- Gapdh: Forward: TCCATGACAACTTTGGCATTG; Reverse: CAGTCTTCTGGGTGGCAGTGA;

The qPCR analysis was performed in the StepOnePlus real-time PCR system by SYBR green I dye detection (Vazyme). The final volume of the quantitative PCR amplification reaction is 10 μl, The thermal cycling conditions include pre-denaturation at 95 °C for 30 s, 40 cycles of denaturation at 95 °C for 10 s, and annealing and extension at 60 °C for 30 s. Gapdh is used as an endogenous control to standardize the difference. The melting curve is constructed after the cycle to ensure that no non-specific products are present. Quantification was performed by using the normalized *Ct* (cycle threshold) value of Gapdh *Ct* and analysis using the 2^−ΔΔ*Ct*^ method.

### Immunohistochemistry

As we previously reported [19], we used isoflurane to anesthetize mice and perfuse with saline and 4% paraformaldehyde through the ascending aorta. After perfusion, the cervical skin and the C3-4 spinal cord segment were taken and fixed overnight. The spinal cord section (30 μm, free-floating) was cut in a cryostat and subjected to immunohistochemical treatment. Firstly, block the sections with 2% goat serum at room temperature for 1 h, then incubated overnight at 4 °C with the primary antibodies: Iba-1 antibody (1:1000, rabbit; Wako, catalog: 019-19741), GFAP antibody (1:1000, mouse; Millipore Bioscience Research reagent, catalog: MAB360), using PBS repeatedly wash 3 times then Cyanine 3-anti-mouse (1:1000, Jackson ImmunoResearch, catalog: 115-165-003) or Alexa 488-anti-rabbit (1:1000, Jackson ImmunoResearch, catalog: 111-545-003) mixture incubated for 2 h at room temperature. Check the stained section with the Nikon fluorescence microscope, and capture the image with a CCD Spot camera. Six spinal cord slices were collected from each mouse for quantitative immunofluorescence. The blind observer using ImageJ software to measure the fluorescence intensity of the superficial dorsal horn of the spinal cord (laminae I-III).

### Cells culture and migration

Muse cells were generated from human-MSC, which was provided from Genesis Stem Cell Regenerative Medicine Engineering Co., LTD, China, by 8-h incubation of trypsin, followed by vortexing at 2,200 rpm for 3 min and centrifugation at 740×*g* for 15 min. The cells were cultured at 37 °C in α-minimum essential medium (α-MEM) containing 10% (volume/volume) FBS and 0.1 mg/mL penicillin–streptomycin solution [20, 21]. After 2 days of culture, when the cells cover 90% of the culture dish, using 0.05% trypsin to digest cells, centrifugation at 1000 rpm to collect the Muse cells for subsequent experiments. The supernatant of Muse cells was collected and made into powder with vacuum freeze-drying. The powder was dissolved in PBS for subcutaneous injection or in the HaCat cell culture medium for culture. The solution used to dissolve the freeze-dried powder was half of the initial volume. HaCat cells were purchased from Shanghai Zhongqiao Xinzhou Biotechnology Co., Ltd. Cells were maintained at 37 °C in Dulbecco's Modified Eagle Medium (DMEM) containing 10% (vol/vol) FBS and 0.1 mg/mL penicillin–streptomycin solution. We use the ibidi culture–insert for wound healing assays to observe the effect of the supernatant of Muse cells on the migration of HaCat cells. First of all, a physical gap was created within a cell monolayer, we monitor and record the process of cell migration to the gap by taking pictures at 0 h, 6 h and 12 h. The gap closure rate was analyzed by using ImageJ software.

### Cells labeling and EdU labeling of cultured cells

Muse cells were incubated with Vybrant CM-Dil (Molecular Probes, Life Technologies) according to the instructions. In brief, a 1000-fold dilution of dye was added to the resuspended Muse cells suspension, then placed in the incubator incubated for 15–20 min, centrifuge for 5 min at 1000 rpm. Washing repeatedly 2–3 times to wash the excess dye to obtain the labeled Muse cells. HaCat cells were grown on glass coverslips in DMEM containing with 10% FBS and 1%PS. EdU was added to the culture medium at a concentration of 10 nM to 10 μM for 30 min. Later the cells were washed 2 to 3 times with PBS, permeabilized and fixed. The next steps are the same as immunohistochemistry.

### Statistical analyses

All data were expressed as mean ± SEM. One-way and Two-way ANOVA followed by the Bonferroni test was used to analyze the behavioral data. Student’s t-test was applied only 2 groups were to be compared. The criterion for statistical significance was *p* < 0.05.

## Results

### Muse cells alleviate scratching behavior in AD mice

Chronic itching is a sensation that triggers the desire to scratch. Repeated applications of 2,4-dinitrofluorobenzene (DNFB) were used to induce AD in a mouse model of annoying pruritus. Scratching behavior was recorded for 30 min every time on different days, as shown in a schematic diagram (Fig. 1A). Following the last treatment of DNFB, we subcutaneously injected 2 × 10^5^ Muse cells (Fig. 1B) or phosphate-buffered saline (PBS) into the neck of DNFB-stimulated mice. After the model was established, the skin on the backs of the mice became red, and the number of robust scratches increased compared with baseline, causing skin damage and bleeding. The mouse skin and hair around the experimental site were slightly reduced due to the excessive scratching [16]. Muse cell administration quickly reversed the itching and scratching behaviors after the last administration of DNFB, the redness of the skin on the backs of the mice was relieved, and the wounds caused by scratching gradually recovered compared to those of mice treated with PBS alone. The scratching behavior of mice in the PBS group did not decrease and even slightly increased due to the developmental period of the pruritus model; the mice appeared irritable and behaved abnormally compared with mice in the Muse cells treatment group. The effects of Muse cells lasted for 5 days after the final administration, after which there was no significant difference in either group (Fig. 1C). On day 5–10, both groups of AD mice gradually recovered from scratching behavior, and in Muse-cell-treated group, the number of scratches returned to the baseline on day 9. However, the number of scratches in vehicle group was still much higher than that in the naive group on day 10. These results suggest that Muse cells can alleviate scratching behavior in AD mice.

To observe the migration of Muse cells after subcutaneous injection in the back skin, we used CELL TRACKER dye to label Muse cells, which were injected subcutaneously into the model site in situ. Day 1, 3, 5, 7, 9 and 11 post injection, the mice were perfused with paraformaldehyde, skin tissues were collected, and frozen sections were obtained. The immunofluorescence results showed that after subcutaneous injection, a large number of Muse cells were present in the skin lesions and slightly spread around the lesions, and some Muse cells had migrated into the dermis. Immunofluorescence analysis showed that the Muse cells gradually spreading from the injection site throughout the entire damaged skin site. We hypothesize that due to the increase in inflammatory factors in the skin, chemokines may be secreted, inducing Muse cells to spread to the damaged area. The number of Dil-labeled Muse cells gradually declined, with only a few cells observable on day 11 after injection (Fig. 1D).

### Muse cells have the potential for skin regeneration and reduce the expression of inflammatory cytokines in the spinal cord and skin

**Fig. 2:**
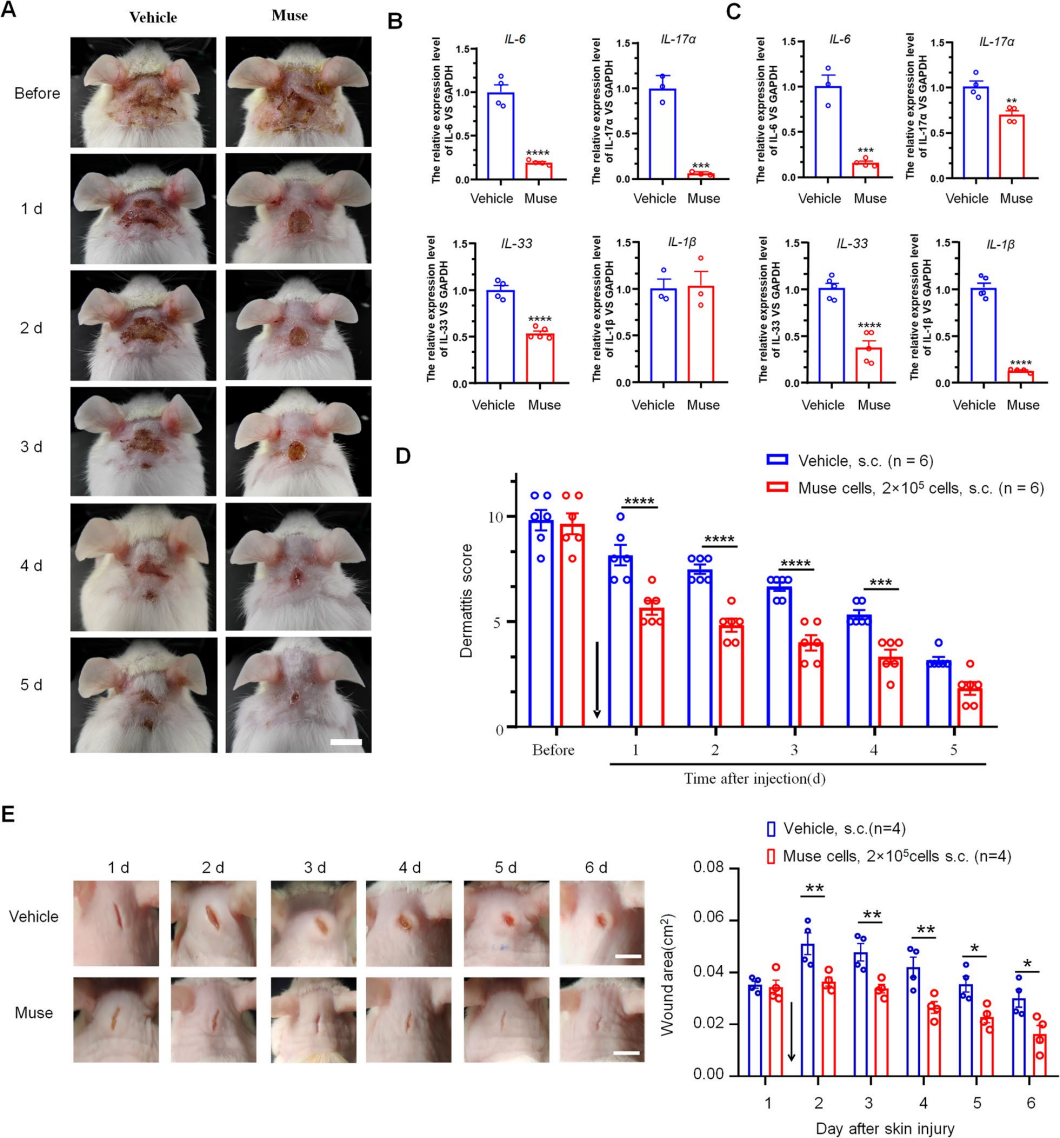
Muse cells promote the regeneration of skin lesions in AD mice and reduce the expression of spinal and skin proinflammatory cytokines. **A** The histological changes in the skin of AD mice were recorded by photos. After the administration of DNFB, erythema of the back skin and edema in the ear pinna were markedly increased. Skin lesions on the back of the neck were consecutively photographed 5 days after subcutaneous administration of PBS (vehicle) and Muse cells in AD mice for pathological evaluation. Compared with those in the vehicle group, changes in the symptoms of wound healing, bleeding, erosion and dryness on the back skin in the Muse group were significantly relieved. Scale bar = 1 cm. Real-time PCR analysis of spinal **B** and skin **C** levels of the cytokines *IL-6, IL-17α, IL-33*, and *IL-1β.* **B** Spinal levels of the cytokines *IL-6, IL-17α*, and *IL-33* were significantly reduced in response to Muse cell treatment. The cytokine *IL-1β* was not different between the two groups. **C** Skin levels of the cytokines *IL-6, IL-17α, IL-33*, and *IL-1β* were significantly reduced in response to Muse cell treatment. The results were normalized to *Gapdh* and are shown as ratios relative to vehicle (PBS-treated) mice under chronic itch conditions. Two-tailed Student’s t-test; ***p < 0.001, **p < 0.01 *versus* vehicle group; *n* = 3–5 mice per group. The data are presented as the mean ± s.e.m. **D** The dermatitis score shows the therapeutic effect of Muse cells or PBS on AD mice. Muse cell treatment had a significant effect on the recovery of diseased skin. Similar to the behavioral results, the therapeutic effect of Muse cells lasted for 4 days. Two-way ANOVA followed by the Bonferroni test; ***p < 0.001*versus* vehicle group; *n* = 6 mice per group. The data are presented as the mean ± s.e.m. Arrow indicates the time of Muse injection. **E** A skin wound injury model was used to check the effects of Muse cells on skin wound healing. Compared with the control group, subcutaneous injection of Muse cells could significantly promote skin wound healing. Two-way ANOVA followed by the Bonferroni test; ***p < 0.001*versus* vehicle group; *n* = 4 mice per group. Arrow indicates the time point of Muse cells or PBS injection. The data are presented as the mean ± s.e.m

In CP diseases, scratching behavior is very common, and annoying, constant scratching causes deficiencies in many stratum corneum (SC) components that contribute to skin barrier function [17, 18]. As the itching intensified, the number of scratches also increased, which eventually led to aggravated skin damage, forming a vicious cycle of itching-scratching-skin damage. Using DNFB, erythema of the back skin and edema in the ear pinna were markedly increased. We examined the effects of subcutaneously administered Muse cells compared to PBS treatment. The histological changes in the skin were evaluated by imaging for 5 days after subcutaneous administration of Muse cells and PBS. PBS did not improve the severity of skin lesions in AD mice, but Muse cells significantly alleviated skin damage (Fig. 2A). Observing the faces and ears of the mice and the degree of back bleeding, erosion and dryness, it could be seen that there was a significant difference between the PBS and Muse treatment groups; Muse cell-treated mice showed better and faster therapy effects than PBS-treated mice, and the time to wound healing and scab shedding was greatly shortened [19]. Further evaluation of dermatitis showed that the effectiveness of Muse cells began on the first day after injection and was maintained until the fourth day (Fig. 2D). Our results showed that Muse cells play an important role in protecting the skin in chronic itching diseases. This treatment can not only break the vicious cycle but also promote the regeneration of diseased skin. The skin regeneration in the AD mice may either benefit from the positive effects of Muse cells or the reduction of scratching. To confirm the effects of Muse cell on skin recovery, we used a skin wound injury model, which did not induce scratching behavior. Compared with the control group, subcutaneous injection of Muse cells could significantly promote skin wound healing (Fig. 2E). These results indicated that Muse cells can promote skin regeneration.

During the establishment of the DNFB model, we observed that erythema of the back skin and edema in the ear pinna were serious, and the skin was damaged and eroded due to severe scratching. Related literature reports that inflammatory factors also play an important role in AD [20], and so we hypothesized that the vicious cycle of itching-scratching lesions was exacerbated by inflammation. To explore the role of inflammatory substances in the spinal dorsal horn and damaged skin in the CP model, we evaluated changes in the inflammatory cytokines IL-17α, IL-6 IL-33, and IL-1β. Real-time PCR analysis showed that after the AD model was established, compared with that in the vehicle (PBS-treated) group, the expression of IL-17α, IL-6, and IL-33 mRNA in the Muse cell group was significantly decreased in the spinal cord, while the spinal-released cytokine IL-1β was not different between the two groups (Fig. 2B). In the skin sample, real-time PCR analysis of the skin-released cytokines IL-17α, IL-6 IL-33, and IL-1β revealed that the increase in proinflammatory cytokines was markedly decreased by Muse cells (Fig. 2C). These results indicate that Muse cells may play a therapeutic role by regulating inflammatory factors related to CP.

### DNFB-induced glial cell activation is reduced in Muse cell-treated mice

**Fig. 3:**
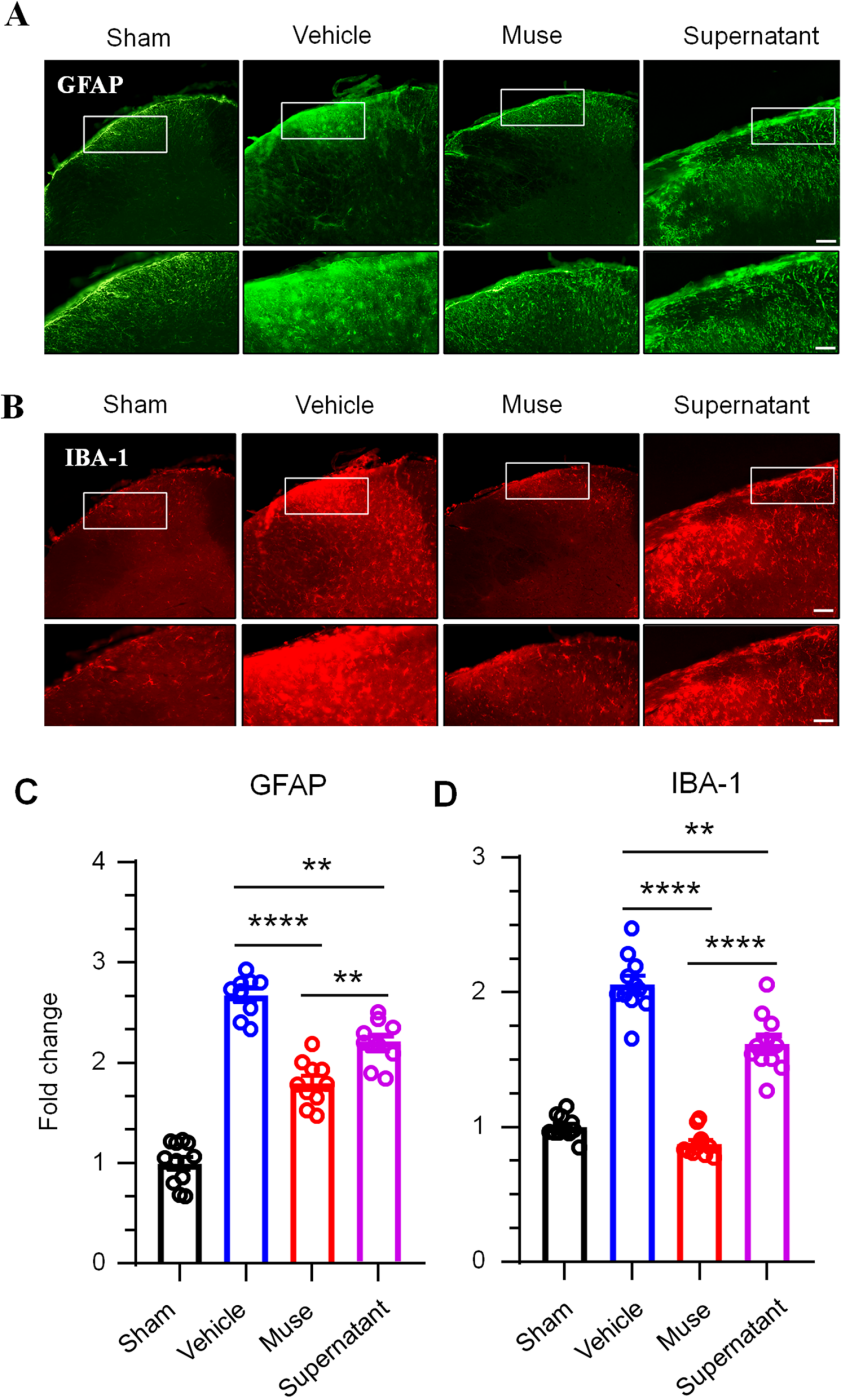
The effect of Muse cells on glial cell activation in the spinal dorsal horn. **A**, **B** Four DNFB administrations on the back skin induced glial cell activity in the cervical spinal cord. Immunohistochemistry was used to examine the activation of astrocytes and microglia in the dorsal horn of the cervical spinal cord (C3-C4). The expression of GFAP and IBA1 in the spinal cord increased significantly after DNFB administration, indicating that the CP model caused the activation of astrocytes and microglia. The activation of microglia and astrocytes in the Muse cell and supernatant treatment group were significantly suppressed. Compared with the supernatant, Muse cells had stronger inhibitory effect. Scale bar = 100 μm in the top and 50 μm in bottom panels. **C**, **D** Quantitative analysis of GFAP, IBA-1, and the immunofluorescence intensity (fold change relative to the naive control) in the spinal dorsal horn of naive and PBS and Muse cells and Muse cell supernatant-treated mice. One-way ANOVA followed by the Bonferroni test. ***p < 0.001 *versus* naive; *n* = 11 slices from 5 mice per group. The data are presented as the mean ± s.e.m

The literature shows that glial cells in the spinal cord play a key role in the maintenance of CP [21–23]. To determine whether the microglia and astrocytes in the spinal cord of AD model mice can be activated, we measured the expression of astrocytic and microglial markers in the C3-C5 spinal cords of naive, PBS-treated, Muse-treated and supernatant of Muse cells-treated mice 3 days after injection by immunohistochemistry. The results showed that administrations of DNFB on the back skin led to upregulation of the astrocytic marker GFAP and the microglial marker IBA-1, as well as morphological changes in astrocytes and microglia in the cervical spinal cord. All of these glial changes were attenuated by the treatment of either Muse cells or their supernatant, though Muse cells had stronger inhibitory effects (Fig. 3). The expression of GFAP in the Muse cell group was relatively increased, and IBA-1 was not significantly different from that in the naive group indicate astrocytes play a key role in the duration of itching.

### Muse cell supernatant has a positive regulatory effect on wound healing and promotes HaCat cell proliferation

**Fig. 4:**
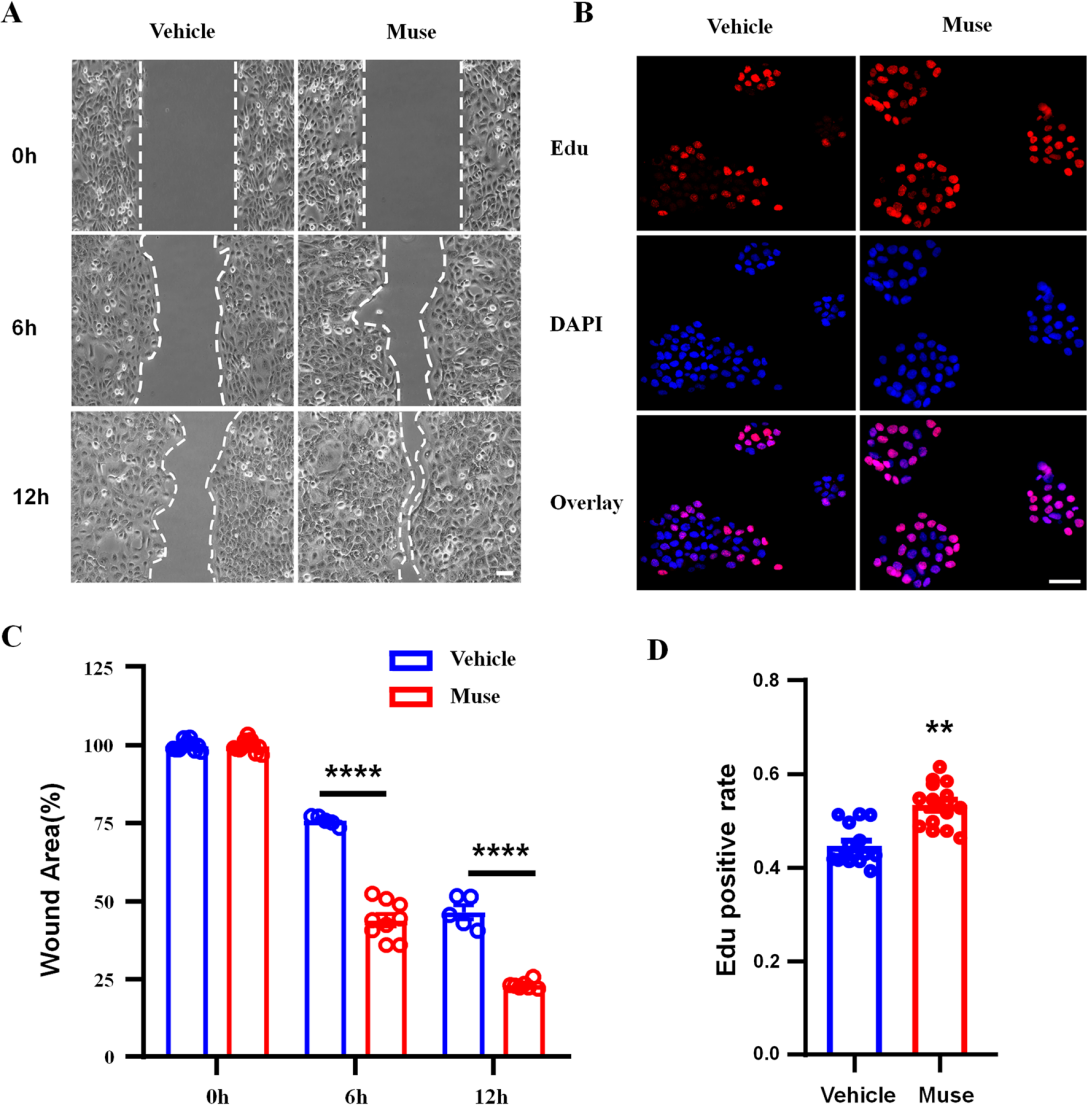
The effects of Muse cell supernatant on wound healing and the proliferation of keratinocytes. **A** Muse cell suspensions were inoculated into the plug-in chamber, and then the cells were cultured for 1 day. Cell migration was observed, and photos were taken at 0 h, 6 h and 12 h in the different groups. The area at 0 h was used as a control. The results showed that compared with that in the control group, the migration rate of HaCaT cells in the Muse cell supernatant treatment group was significantly accelerated at 6 and 12 h. **B** HaCaT cells were cultured on glass coverslips, and then Muse cell supernatant was added and incubated for 30 min. Cell proliferation was observed, and photos were obtained after immunofluorescence staining. The proliferation of HaCaT cells was slightly accelerated in the Muse cell supernatant treatment group compared with the vehicle group. **C** Statistical analysis of HaCaT cell migration areas. Two-way ANOVA followed by the Bonferroni test; ***p < 0.001*versus* 0 h; *n* = 9 slices in each group. The data are presented as the mean ± s.e.m. **D** Statistical analysis of EdU analysis of HaCaT cell proliferation. Two-tailed Student’s t-test; *p < 0.05 *versus* vehicle group; *n* = 15 slices from 5 cultures per group. The data are presented as the mean ± s.e.m

Keratinocytes (HaCat cells) account for approximately 95% of total human epidermal cells and maintain the biochemical and physical integrity of the skin by secreting large amounts of high molecular weight proteins such as cytokeratin and mucopolysaccharides. Under normal steady-state conditions, the epidermis can protect the body from external damage [24]. Due to the repeated scratching of patients with chronic itching, the epidermal barrier weakens, breaks and disappears, causing greater damage to patients. We used the Ibidi culture insert for wound healing assays to observe the effect of Muse cell supernatant on the migration of HaCat cells. As shown in Fig. 4A, after removing the middle insert plug-in, we used an inverted microscope to take photos at three different times (0 h, 6 h, and 12 h) to observe the process of cell migration to the middle of the gap. The area at 0 h was used as the control. The results showed that compared with that in the control group, the migration rate of HaCat cells in the Muse cell supernatant treatment group significantly increased. Clear differences between the two groups were observed at 6 and 12 h (Fig. 4C). This result indicates that Muse cell supernatant plays an active role in promoting wound healing in vitro [19].

The healing of skin wounds is always accompanied by the proliferation of keratinocytes (HaCat cells). In vivo, we observed that Muse cells could increase the speed of wound healing. We have proven that Muse cell supernatant can promote the migration of keratinocytes in vitro. Furthermore, we hypothesized that this supernatant would also affect the proliferation of HaCat cells. Using the EdU kit, we separately tested the proliferation of HaCat cells in the Muse cell supernatant-treated group and the untreated group (vehicle), and the results showed that compared with the vehicle, Muse cell supernatant promoted the proliferation of keratinocytes and further accelerated wound healing (Fig. 4B). The statistical analysis of the cell proliferation rate further confirmed these findings (Fig. 4D). Thus far, we have confirmed the reparative effect of Muse cells in vivo and in vitro.

### The protective effect of Muse cell supernatant on LPS-induced HaCat cells

**Fig. 5:**
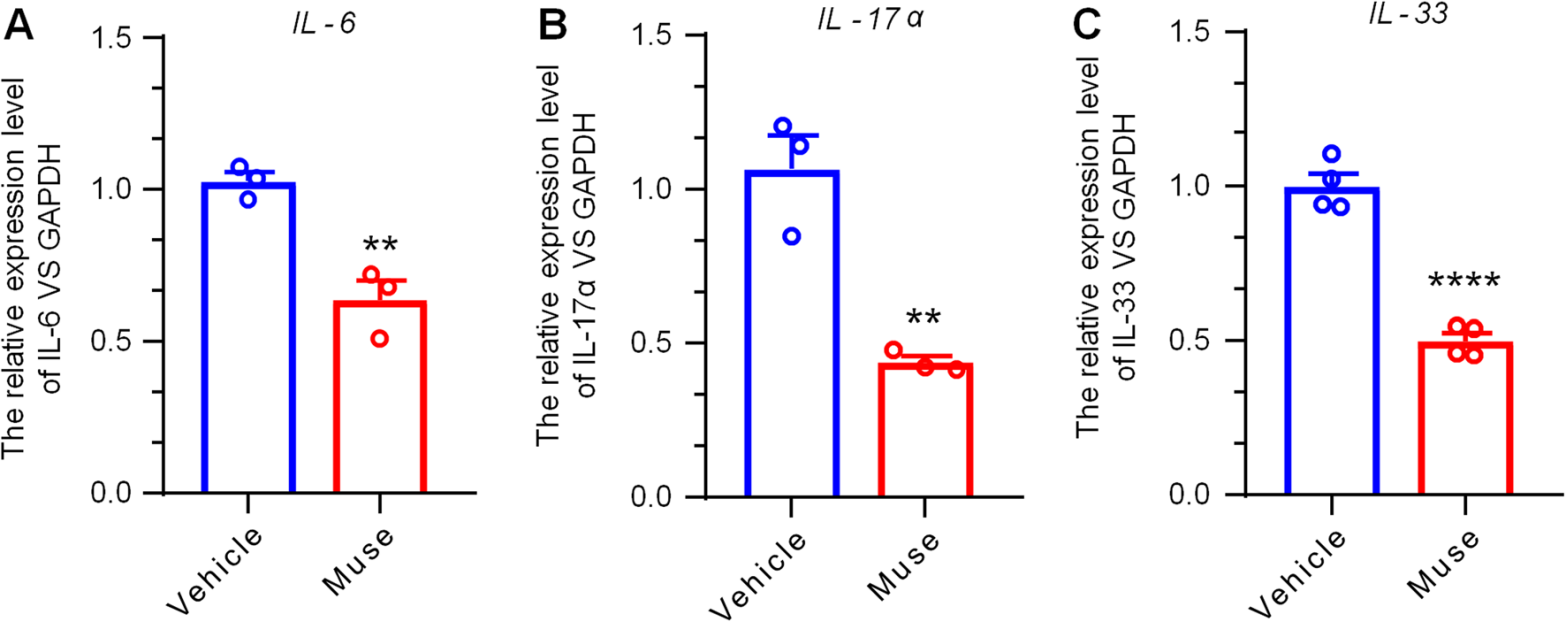
The protective effects of Muse cell supernatant on LPS-induced HaCaT cells. **A**–**C** The mRNA expression of *IL-17α*, *IL-6* and *IL-33* in HaCaT cells was measured by real-time PCR. Each inflammatory factor was subjected to three independent repeated analyses, and HaCaT cells without Muse cell supernatant were used as the vehicle group. The results were normalized to Gapdh. The results showed that under inflammatory infection conditions, the increase in proinflammatory cytokines was powerfully inhibited by pretreatment with Muse cell supernatant. Two-tailed Student’s *t*-test; ***p < 0.001, **p < 0.01 *versus* vehicle group; *n* = 3–4 cultures per group. The data are presented as the mean ± s.e.m

LPS is the main component of gram-negative bacterial membranes and is one of the most common inflammatory stimulants. We used LPS to establish a human keratinocyte (HaCat) bacterial infection and inflammation model to investigate whether Muse cell supernatant could downregulate the inflammatory factors IL-17α, IL-6 and IL, as shown in the in vivo experiment [25]. By inhibiting inflammation and reducing patient scratching behaviors, the patient's skin biochemical and physical barrier function can be restored to achieve treatment efficacy. Our results showed that the expression of the proinflammatory cytokines Il-6, Il-17α and Il-33 in Muse cell supernatant-pretreated keratinocytes was significantly inhibited compared with that of cells that were not pretreated (Fig. 5A). Thus far, we have demonstrated in vivo and in vitro that Muse cells have a robust ability to regulate inflammation.

## Discussion

AD is a common inflammatory skin disease characterized by recurrent eczema skin lesions and severe itching, which heavily affects patient quality of life and sleep quality [26]. The pathogenesis and etiology of AD are complicated and may involve intense interactions between keratinocytes, immune cells, and skin neurons [27]. The pathophysiology of clinical AD is unclear, so there is no universally effective treatment for CP caused by AD. There are a variety of clinical treatments, such as emollients, opioids, anti-inflammatory treatments, and conventional systemic treatments, to help relieve the disturbing symptoms of scratching [1, 27]. The existing methods are not effective for many patients, and the side effects of drugs are significant. Therefore, there is urgent to find new and effective treatments for AD.

Our study is the first to report that Muse cells can alleviate the scratching behavior of mice with AD, promote the repair of damaged skin, and provide a new strategy for the treatment of chronic itching. The applications of mesenchymal stem cells in the repair of tissue damages from injury or diseases have been greatly increased. Adipose-derived mesenchymal stem cells (AD-MSCs) are one important and widely used type among them. They can be used alone in autologous applications [28] for the treatment of soft tissue defects and chronic wounds [29, 30], or can be employed in conjunction with dermal substitute scaffolds and platelet-rich plasma. In addition, when combined with SVF-enhanced autologous fat grafts, they have been applied in scar treatment from breast surgery, facial rejuvenation, buttock augmentation, Romberg syndrome, and liposuction sequelae [31]. Muse cells are endogenous pluripotent stem cells derived from MSCs that have reparative properties. Both allogeneic transplantation and xenotransplantation of Muse cells can promote functional recovery [9]. The study of Muse cells in CP has not yet been reported, so we conducted research on Muse cells. In our study, we first established a chronic itch model induced by DNFB and then subcutaneously injected Muse cells in situ to explore whether the symptoms of chronic itch could be alleviated. Our results showed that in the CP model, the number of scratches on mice increased significantly compared with that in the untreated group. After treatment with Muse cells, the scratching behavior of mice greatly decreased. We observed behavioral changes on the first day after injection, and the treatment effect continued until the fourth day and was still significantly different.

The SC is the basis of the normal skin barrier and is composed of keratinocytes supported by the lamellar matrix. The basic function of the SC is to form a physical and biological barrier in the skin, prevent the loss of transdermal evaporative water, and provide a site for the colonization of nonpathogenic bacteria [32]. Due to the presence of the vicious cycle of itching-scratching-skin damage in patients with chronic itching, the SC is damaged, and its barrier function is destroyed. The destruction of the physical barrier leads to not only increased moisture penetration of the skin but also to a higher rate of bacterial infection. The pH of the SC changes, and the loss of ceramide metabolites and other functional molecules with antibacterial effects will further destroy the antibacterial function and physical barrier effect of the skin [33–35]. In the mouse model of CP, skin erosion and bleeding on the back of the neck were significant, damaging the barrier function of the skin. After treatment with Muse cells, the dermatitis score of the neck skin of mice was significantly relieved, and wound healing of the skin lesion was also greatly accelerated.

Glial cells are not only involved in neuropathic pain but also related to CP. The central nervous system is mainly composed of astrocytes and oligodendrocytes. CP has been reported to activate central glial cells and increase activated astrocytes [36–40]. To explore the effect of glial cells on the CP model, we examined the activation of astrocytes in the cervical spinal dorsal horn of mice, and the immunofluorescence results showed that in the DNFB-induced CP model, astrocytes were greatly activated. In the group treated with Muse cells, the number of activated astrocytes in the spinal dorsal horn were sharply reduced but was still higher than that in the naive group. We hypothesize that due to the long period of the DNFB model, the increased scratching behavior of mice led to the longterm activation of astrocytes. The scratching behavior of mice in the Muse cell treatment group was greatly reduced, which resulted in a decrease in activated astrocytes in the spinal cord. The mice in the naive group had no obvious scratching behavior, so astrocytes were not activated [20, 22]. Microglia are another type of glial cell that continuously monitors danger factors in our bodies. When the internal environment is at risk, microglia will change from a monitoring cell type to an activated form, specifically in response to the stimulated area [41]. Our results showed that microglial activation was rapid and transient. Compared with that in the PBS group, microglial activation in the Muse cell treatment group was significantly reduced, and there was no difference compared with that in the naive group.

Chronic itching is a symptom of many types of skin inflammation, and the treatment of CP is always accompanied by the treatment of skin inflammation [42]. Inflammatory cells release mediators that affect the corresponding receptors on sensory neurons, leading to aggravation of the inflammatory response; furthermore, skin inflammation will lower the threshold of itching stimulation, forming a positive feedback loop [43]. In our study, the expression levels of IL-17α, IL-6, and IL-33 in the spinal dorsal horn and back skin of the AD model group were upregulated. After treatment with Muse cells, the level of inflammatory factors in model mice was significantly decreased. These results indicate that CP can increase the release of inflammatory substances; however, Muse cells can reduce the inflammatory response and inhibit the itching behavior of mice. Thus far, we have verified in vivo that Muse cells can inhibit the secretion of inflammatory factors, restore skin damage to maintain the physical and biological barriers of the skin, alleviate scratching behavior and reduce the activation of astrocytes and microglia.

Keratinocytes were used for in vitro experiments, and we used cell migration assays to simulate wound healing. Muse cell supernatant was added to the culture dishes, and the cell migration rate was observed. The results showed that Muse cell supernatant could accelerate the migration of cells from the edges of the scratches to the center. Compared with that in the control group, the therapeutic effect was significantly different at 6 and 12 h. We also designed an in vitro inflammatory stimulation model to observe the anti-inflammatory effects of Muse cells. LPS was used to stimulate keratinocytes to induce inflammation. Muse cell supernatant was added to protect keratinocytes. The results showed that Muse cells could reduce the expression levels of the inflammatory factors IL-17α, IL-6, and IL-33 in keratinocytes in vitro. The in vivo and in vitro experimental results proved that Muse cells could effectively improve the scratching behavior of chronic itching, slow the development of inflammation, and promote the recovery of damaged skin. This discovery led us to hypothesize that after subcutaneous injection of Muse cells in situ, these cells secrete growth factors or exosomes, which change the local microenvironment of the skin lesion and suppress the secretion of inflammatory factors, thereby reducing the scratching behavior of chronic itching and participating in the recovery of skin lesions.

Obtaining Muse cells is relatively easy, which makes this therapy convenient for clinical use. For clinical research, mesenchymal stem cells, especially adipose stem cells combined with tissue engineering, have been applied in wound healing [28, 44], autologous transplantation [45], and scar repair [46] [47, 48]. These methods and clinical research have provided us with the inspiration and the next direction for the research. Local injections under the skin, the approach we selected, had been demonstrated effective. In the future, we will try to combine Muse cells with tissue engineering by retaining the cells in a hydrogel, which will be implanted directly in vivo for tissue repair and wound healing. We have not yet examined the possible mechanism by Muse cells treat CP. We hypothesize that this may be due to immune regulation or exosomes secreted by Muse cells that change the local microenvironment of the injection site and promote skin regeneration. Further study is needed to confirm this conclusion.

## Conclusions

In summary, our study showed that Muse cells could alleviate chronic itching skin damage, relieve annoying scratching behavior, and inhibit the activation of glial cells in the spinal dorsal horn. Although the specific mechanism has not been thoroughly studied, Muse cell administration is still a new idea and method for the treatment of chronic itching.

## Data Availability

Data and materials will be provided upon private request.

## Abbreviations

AD: Atopic dermatitis
DNFB: 2,4, Dinitrofluorobenzene
GAPDH: Glyceraldehyde-3-phosphate dehydrogenase
GFAP: Glial fibrillary acidic protein
HaCat: Human immortalized keratinocytes
LPS: Lipopolysaccharide
Iba1: Ionized calcium binding adapter molecule1
LTT: Long-term trypsin incubation
Muse cells: Multilineage differentiating stress enduring cells
MSCs: Mesenchymal stem cells
